# Modeling Pathogenic Mutations of Human Twinkle in *Drosophila* Suggests an Apoptosis Role in Response to Mitochondrial Defects

**DOI:** 10.1371/journal.pone.0043954

**Published:** 2012-08-28

**Authors:** Alvaro Sanchez-Martinez, Manuel Calleja, Susana Peralta, Yuichi Matsushima, Rosana Hernandez-Sierra, Alexander J. Whitworth, Laurie S. Kaguni, Rafael Garesse

**Affiliations:** 1 Departamento de Bioquímica, Instituto de Investigaciones Biomédicas “Alberto Sols” Universidad Autónoma de Madrid-Consejo Superior de Investigaciones Científicas, and Centro de Investigación Biomédica en Red en Enfermedades Raras, Facultad de Medicina, Universidad Autónoma de Madrid, Madrid, Spain; 2 Instituto de Investigación Santitaria Hospital 12 de Octubre (i+12), Madrid, Spain; 3 Centro de Biología Molecular “Severo Ochoa” Universidad Autónoma de Madrid-Consejo Superior de Investigaciones Científicas, Madrid, Spain; 4 Department of Biochemistry and Molecular Biology and Center for Mitochondrial Science and Medicine, Michigan State University, East Lansing, Michigan, United States of America; 5 Department of Biomedical Sciences, MRC Centre for Developmental and Biomedical Genetics, University of Sheffield, Sheffield, United Kingdom; University of Texas Health Science Center at San Antonio, United States of America

## Abstract

The human gene *C10orf2* encodes the mitochondrial replicative DNA helicase Twinkle, mutations of which are responsible for a significant fraction of cases of autosomal dominant progressive external ophthalmoplegia (adPEO), a human mitochondrial disease caused by defects in intergenomic communication. We report the analysis of orthologous mutations in the *Drosophila melanogaster* mitochondrial DNA (mtDNA) helicase gene, *d-mtDNA helicase*. Increased expression of wild type *d-*mtDNA helicase using the UAS-GAL4 system leads to an increase in mtDNA copy number throughout adult life without any noteworthy phenotype, whereas overexpression of *d-*mtDNA helicase containing the K388A mutation in the helicase active site results in a severe depletion of mtDNA and a lethal phenotype. Overexpression of two *d-*mtDNA helicase variants equivalent to two human adPEO mutations shows differential effects. The A442P mutation exhibits a dominant negative effect similar to that of the active site mutant. In contrast, overexpression of *d-*mtDNA helicase containing the W441C mutation results in a slight decrease in mtDNA copy number during the third instar larval stage, and a moderate decrease in life span in the adult population. Overexpression of *d-*mtDNA helicase containing either the K388A or A442P mutations causes a mitochondrial oxidative phosphorylation (OXPHOS) defect that significantly reduces cell proliferation. The mitochondrial impairment caused by these mutations promotes apoptosis, arguing that mitochondria regulate programmed cell death in *Drosophila*. Our study of *d-*mtDNA helicase overexpression provides a tractable *Drosophila* model for understanding the cellular and molecular effects of human adPEO mutations.

## Introduction

The majority of cellular ATP is generated by oxidative phosphorylation carried out within the mitochondrial inner membrane. Animal mitochondrial DNA encodes 13 polypeptides involved in OXPHOS, whereas all the factors essential for mtDNA replication are encoded in the nuclear genome [1]. As a consequence, the biogenesis of the OXPHOS system is subject to a highly-coordinated, dual genetic control [2]. Mutations in genes encoding the factors essential for mtDNA replication results in both loss of mtDNA integrity via base substitution mutations, duplications and deletions, and in mtDNA depletion [3].

Autosomal dominant progressive external ophthalmoplegia is a human mitochondrial disease associated with the presence of multiple deletions in the mtDNA [3], [4]. The disease has an adult-onset at 20–40 years of age. Its symptoms include muscle weakness, wasting, exercise intolerance, ataxia, hearing loss, cardiomyopathy and peripheral neuropathy [3]. Most adPEO families carry heterozygous mutations in one of three genes: *ANT1* (adenine nucleotide translocator 1), *POLG* (mitochondrial DNA polymerase), or *C10orf2/Twinkle* (mtDNA helicase) [3], [5], [6], [7].

Whereas the nuclear DNA replication machinery is very complex, the number of factors needed to replicate the mitochondrial DNA is comparatively small [8], [9], [10]. The human gene *C10orf2* encodes the essential mitochondrial replicative DNA helicase, Twinkle. Twinkle shares high homology with the bacteriophage T7 gene 4 protein (T7 gp4), which contains both helicase and primase catalytic activities located in its carboxyl- and amino-terminal halves, respectively [6]. The amino acid sequence of the T7 gp4 helicase domain is well conserved in Twinkle, but varies substantially in the primase domain. Twinkle co-localizes with mtDNA structures designated as mitochondrial nucleoids [6], and it has been shown to modulate mtDNA copy number *in vivo* [11]. Studies in human cell culture and in a transgenic mouse expressing a Twinkle variant with an in-frame duplication of amino acids 353–365 that is analogous to a human adPEO mutation showed the accumulation of multiple mtDNA deletions and mitochondrial dysfunction [12], [13].

*Twinkle* mutations are mainly associated with adPEO, but have recently been linked to SANDO [14] and Infantile-Onset Spinocerebellar Ataxia (IOSCA) [15]. Surprisingly, neither mtDNA deletions nor point mutations have been found, suggesting that IOSCA mutations in *Twinkle* affect mtDNA stability in a physiological and tissue specific manner.

Recently, a novel protein with nuclease/helicase activity localized within the mitochondria has been described and designated as DNA2. In humans, hDNA2 forms a complex with the mitochondrial DNA polymerase to stimulate its DNA polymerase activity. Although little is known about this protein, it appears to play a role in processing mtDNA intermediates during replication and repair [16]. However, only a single mtDNA helicase (*d-*mtDNA helicase) has been identified in *Drosophila*.

Several studies have demonstrated that mitochondria play a crucial role in the apoptotic pathway in mammals [17], and thus mutations that affect mitochondrial function such as adPEO, could critically impact this pathway. Despite the fact that almost all of the proteins implicated in cell death are highly conserved in metazoans, the role of mitochondria in apoptosis in *Drosophila* remains controversial [18], [19], [20]. However, one study indicates a significant role for mitochondria in programmed cell death in the fly, where upon apoptosis, the Reaper and HID proteins cause mitochondrial fragmentation and release of cytochrome *c* in both cultured S2 cells and in the developing fly embryo [21]. It is known that factors involved in mitochondrial dynamics play a key role in the segregation of dysfunctional mitochondria [22], and deregulation of these pathways could trigger mitochondrially-mediated apoptosis.

In this study, we overexpressed various adPEO mutations of *d-mtDNA helicase* in *Drosophila melanogaster* to characterize their effects in a tractable animal model, and to analyze the role of dysfunctional mitochondria in apoptosis. Our data show that overexpression of adPEO mutations in the fly causes a severe depletion of mtDNA and as a consequence, an increase in programed cell death. Taken together, our previous [23], [24] and present results give evidence *in vivo* of the crucial role of mitochondria in apoptosis in *Drosophila melanogaster*, providing some clues for the understanding of the pathophysiology of human mitochondrial diseases.

## Results

### A Drosophila Model to Study adPEO Mutations

**Figure 1:**
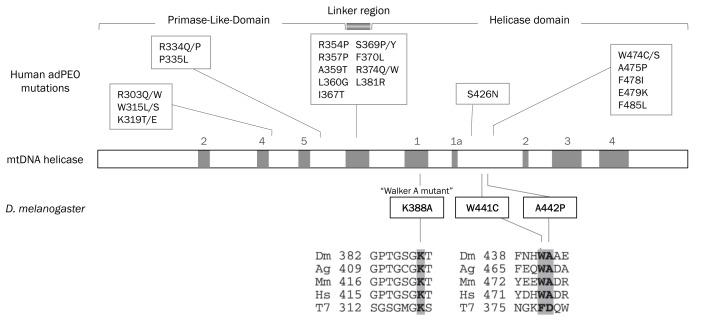
Sequence alignment and location of amino acid substitutions in mtDNA helicase. Schematic diagram of the sequence organization of mtDNA helicase; eight amino acid sequence motifs common to ring primases and helicases are indicated in gray. The bacteriophage T7 gp4 linker region is indicated in the middle of the diagram. Mutations in the human mtDNA helicase gene (*Twinkle/C10orf2*) found in adPEO are shown above the scheme (reviewed in [46]), the orthologous adPEO mutations W441C and A442P in the *Drosophila* mtDNA helicase gene (*d-mtDNA helicase*), and the “Walker A” mutation K388A are shown below the scheme. The underlined mutations indicate autosomal recessive PEO mutations. The positions of mutations in *d-mtDNA helicase* used in this study are shown in bold. Sequence alignment of the regions containing altered amino acids is shown in the lower panel. *Dm*, fly; *Ag*, mosquito; *Mm*, mouse; *Hs*, human; *T7*, bacteriophage T7.

Genomic analysis has identified a number of mutations associated with adPEO in the human mtDNA helicase gene, *C10orf2* [6]. The *Drosophila* genome encodes a highly conserved orthologue in the CG5924 gene, with the two proteins sharing 54.6% identity and 73% similarity (as evaluated by Mobyle Pasteur MATCHER). Most of the amino acid residues mutated in adPEO patients are conserved in the *Drosophila* protein [24], raising the possibility to establish *Drosophila* as a tractable animal model to study them. With this aim, we used the inducible UAS-GAL4 system to express *d-mtDNA helicase* variants containing human adPEO mutations. UAS lines with four versions of the gene were generated: the wild type (wt) version was used as a control, and three versions containing the “Walker A” [27] K388A active site mutation, analogous to K318 in the helicase domain of T7 gp4, and the W441C and A442P mutations, which are analogous to the W474C and A475P mutations found in adPEO patients [6]. These mutations map within the linker region and helicase domain, respectively (Figure 1). Previous results in *Drosophila* S2 cells showed severe mtDNA depletion when *d*-mtDNA helicase containing the K388A or A442P mutations were overexpressed, whereas cells that overexpressed *d*-mtDNA helicase containing the W441C mutation did not show a significant phenotype. In contrast to patients harbouring these mutations, multiple deletions in mtDNA were not observed in cultured fly cells [24].

**Figure 2:**
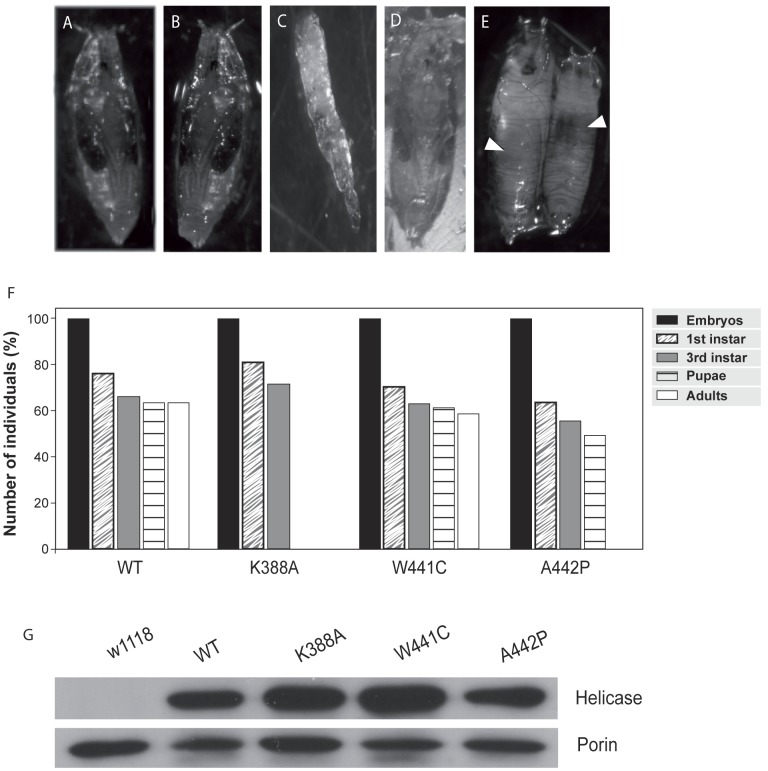
Phenotypes and expression levels of *Drosophila* lines overexpressing mutants of *d-mtDNA helicase*. (A–D); Phenotypes of the lines that overexpress the *d-*mtDNA helicase mutants with a constitutive and ubiquitous driver; (A) *w^1118^;;*, control line*;* (B) *w;;UAS helicase^WT^/da-GAL4,* the phenotype is similar to control line*;* (C) *w;;UAS helicase^K388A^/da-GAL4*, 100% of lethality at third larval instar (D) *w;;UAS helicase^W441C^/da-GAL4*, the phenotype is similar to that of the control line*;* (E) *w;;UAS helicase^A442P^/da-GAL4*, 100% of lethality at pupal stage. White arrowheads show necrotic tissue within the unformed pupae. (F) Quantitation of the viability of the overexpressed lines. (G) Expression levels of *d-*mtDNA helicase. 30 µg of mitochondrial protein from the overexpressing and control lines was used for immunoblot analysis with a polyclonal antibody versus *d-mtDNA helicase*. The *d-*mtDNA helicase protein levels are similar in all of the overexpressing lines whereas the endogenous level in the control line was undetectable. A monoclonal porin antibody was used as an internal loading control.

To characterize the effects of overexpression of the variants of *d-*mtDNA helicase in *Drosophila*, the *daughterless*-GAL4 driver line was used to induce constitutive and ubiquitous expression. Overexpression of the K388A and A442P *d-*mtDNA helicase mutations caused complete lethality in third larval instar and pupal stages, respectively (Figure 2C–E). However, the overexpression of W441C variant and the wild type version did not show any observable phenotype (Figure 2, D). These results were quantified (Figure 2F) and corroborated with multiple independent lines for each mutation. Immunoblot analysis of third instar larvae expressing the *d-*mtDNA helicase variant and control lines showed relatively similar levels of *d-*mtDNA helicase protein among the different lines; in contrast, *d-*mtDNA helicase was undetectable in non-transgenic control animals under the same conditions indicating normally low levels of endogenous expression and the relatively high levels of transgene expression (Figure 2G).

### Constitutive Overexpression of Mutant d-mtDNA Helicase Induces mtDNA Depletion and OXPHOS Impairment in *Drosophila*

**Figure 3:**
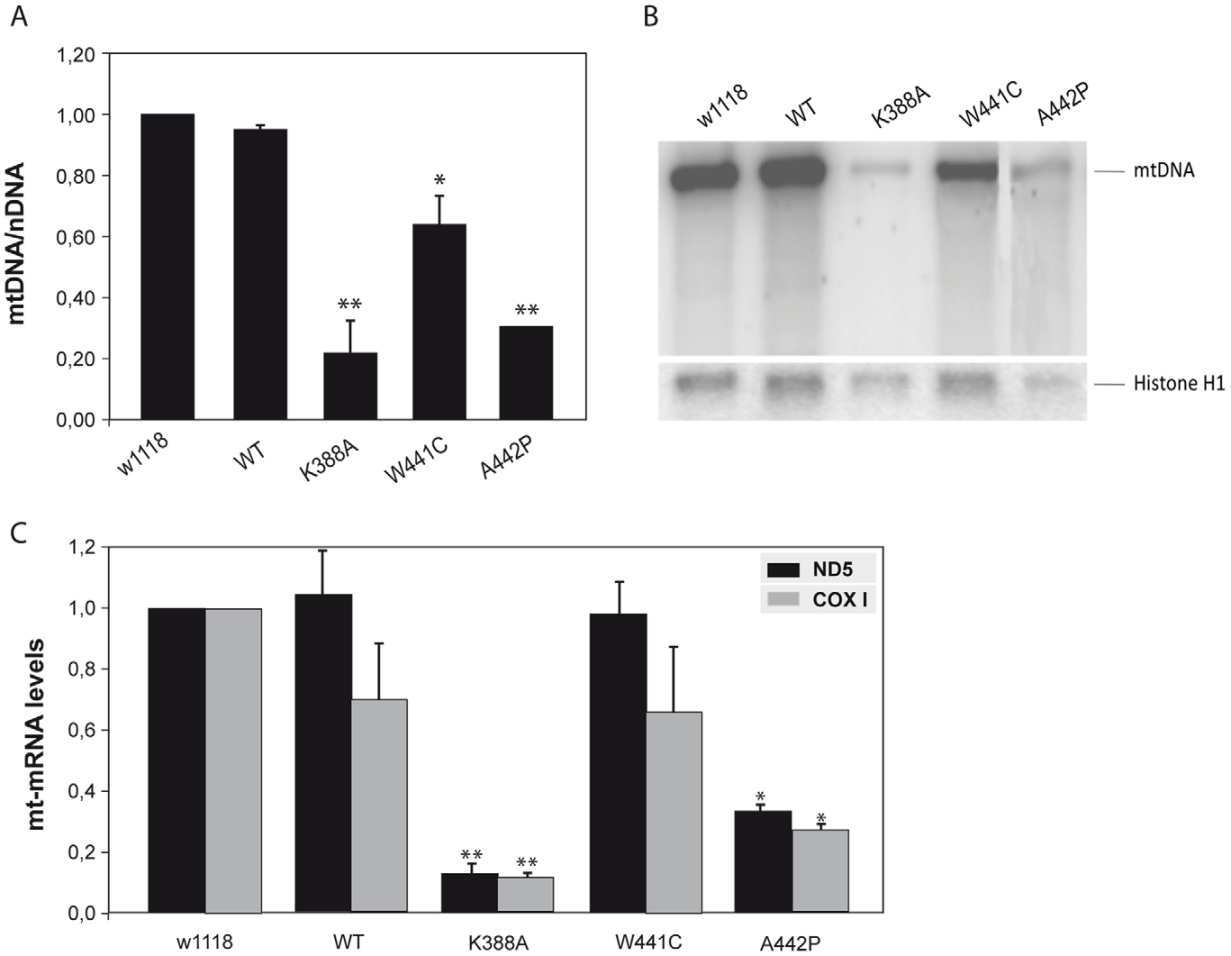
Differential changes in mtDNA copy number and mitochondrial transcript levels. (A) Relative mtDNA copy number was measured by qRT-PCR in third instar larvae of *Drosophila* lines expressing mutant *d-mtDNA helicase*. (B) Total DNA (10 µg) was fractionated in an 0.8% agarose gel, blotted to nylon membrane, and hybridized with radiolabeled probes for the mtDNA to observe multiple deletions. (C) Relative mitochondrial mRNA levels of the *ND5* and *COX I* genes was measured by qRT-PCR. Data represent the mean ± s.d., *P≤0.05, **P≤0.005 as compared with wild-type (*w^1118^*).

**Figure 4:**
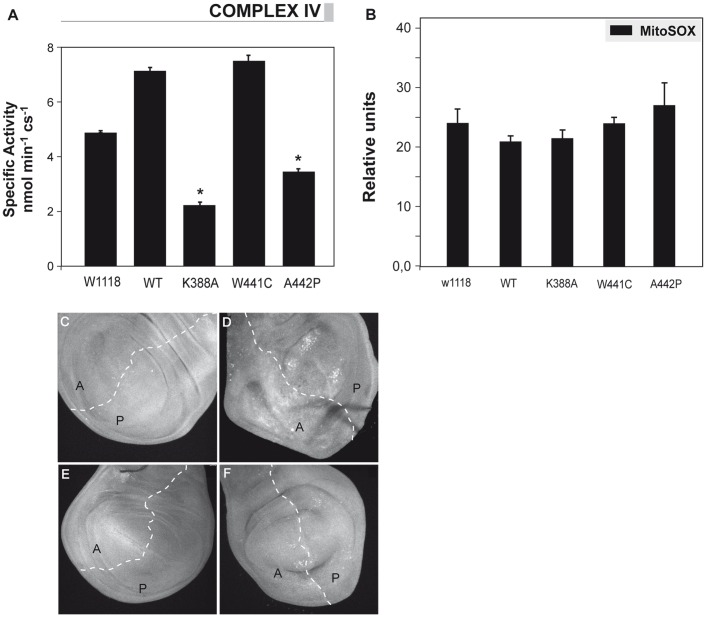
OXPHOS enzymatic activities and ROS levels in *Drosophila* lines expressing mutant *d-mtDNA helicase*. (A) Activities of complex IV were measured in mitochondrial protein extracts obtained from third instar larvae of each genotype expressing the different variants with *da-GAL4*. Data represent the mean ± s.d., *P≤0.05, as compared with wild-type (*w^1118^*), of at least three independent determinations. (B) Imaginal discs from third instar larvae were dissected and digested with trypsin prior to incubation with MitoSox (5 mM). The cell suspension was analysed using a BD Bioscience FACS Vantage SE instrument, and the data were processed with Cell Quest Pro Software. Data represent the mean ± s.d. as compared with wild-type (*w^1118^*). (C–F) Overexpression of *d*-mtDNA helicase with the *engrailed* driver (*en-GAL4*) (posterior compartment, P) in wing imaginal discs, stained with dihydroethidium (DHE 30 µM) that binds superoxide anions. C, *w;en-GAL4;UAS helicase^WT^;* D, *w;en-GAL4;UAS helicase^K388A^*; E, *w;en-GAL4;UAS helicase^W441C^;* F, *w;en-GAL4;UAS helicase^A442P^*.

The effect of overexpression of *d-*mtDNA helicase variants on mtDNA levels was evaluated by quantitative real time PCR analysis. We found a significant decrease in mtDNA copy number in third instar larvae of the lines overexpressing K388A, W441C and A442P mutations as compared with the control lines (Figure 3A). Overexpression of wild type *d-*mtDNA helicase did not cause decrease in mtDNA. However, despite the presence of multiple deletions in mtDNA being a common feature in skeletal muscle in adPEO patients, we found no evidence by Southern Blot (Figure 3B) and long range-PCR analysis (data not shown) of multiple deletions in the mutant or control lines. These results are comparable with data analysing overexpression of *d-*mtDNA helicase active site mutants in *Drosophila* Schneider cells [24]. However, in contrast to the *in vitro* study [24], we also observed significantly decreased levels of mitochondrial transcripts *in vivo* (Figure 3C). To analyze the biochemical consequence of the mtDNA depletion and the decrease in mtDNA-encoded transcripts caused by mutated *d-*mtDNA helicase, we measured the enzymatic activity of cytochrome *c* oxidase (complex IV) in larvae of the control and mutant lines. As expected, we observed a significant decrease in complex IV activity in the K388A and A442P expressing lines (Figure 4A), which showed a clear impairment of the OXPHOS function. Complex IV activity in the W441C line was similar to the control line, consistent with the lack of an apparent phenotype produced by the moderate decrease in mtDNA levels.

To evaluate if the OXPHOS impairment produces any change in the levels of reactive oxygen species (ROS), we employed two different approaches: we used the MitoSox probe that binds specifically to the mitochondrial superoxide anion, and reactive dihydroethidium (DHE), which permeates cell membranes freely, as indicators of cytosolic superoxide production. Neither approach revealed any significant different between the control lines and the overexpression of the *d-*mtDNA helicase variants, even in the presence of OXPHOS impairment (Figure 4B, C). This finding is consistent with that observed in adPEO patients, where no increase of ROS was found.

### Constitutive Overexpression of W441C d-mtDNA Helicase does not Impair OXPHOS Function, but Slightly Reduces Life Span

**Figure 5:**
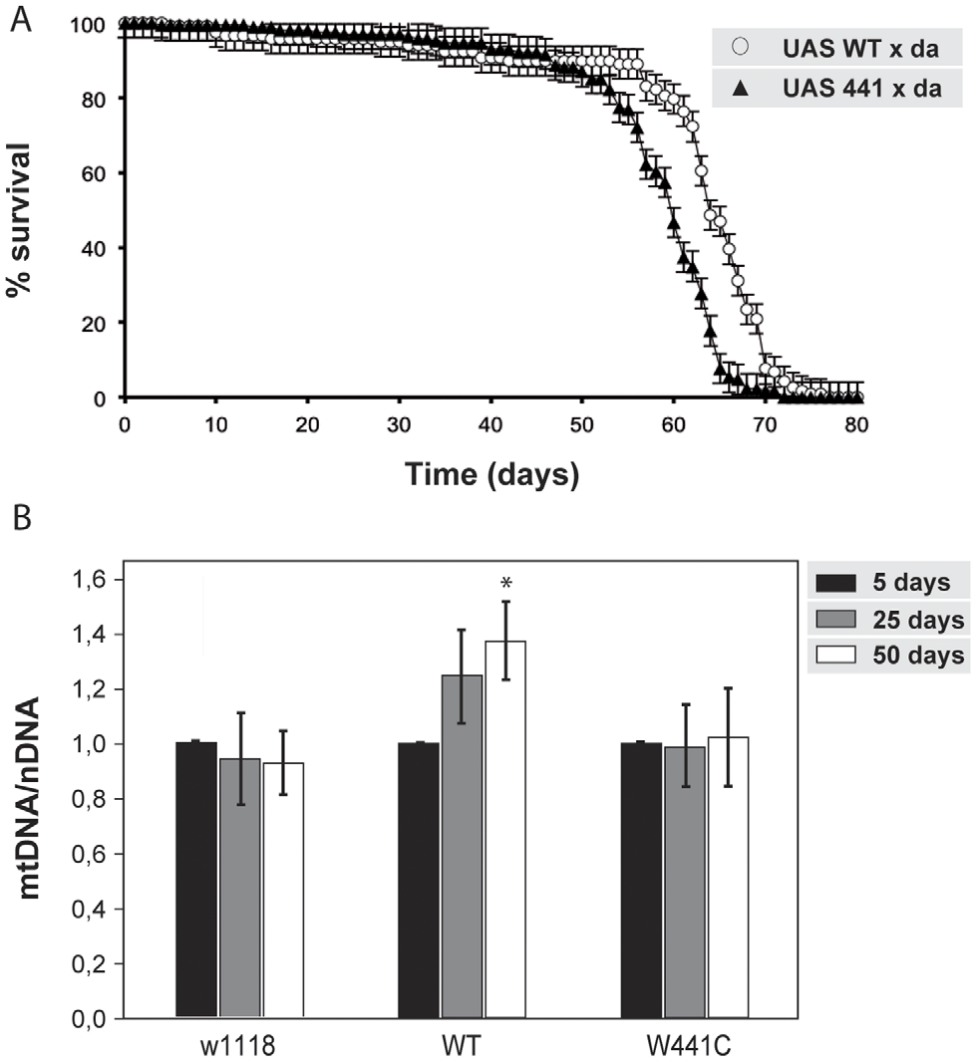
Characterization during adult life of the wild type and W441C mutant *d-*mtDNA helicase. (A) Longevity curve of lines overexpressing the wild type and W441C mutants using a constitutive and ubiquitous driver (*da-GAL4*). 100 adult males (0–2 days old) were collected in an empty tube with standard medium and the number of surviving flies was counted every 2 days. Kaplan-Meier survival curve was performed (p = 0.045, n = 3 biological experiments). (B) Quantitation of mtDNA copy number by qRT-PCR at 5, 25 and 50 days of life in both lines.

Patients carrying adPEO mutations in the *Twinkle* gene have adult onset symptoms between the ages of 20 and 40 years, suggesting that these mutations are only moderately deleterious for enzyme function. Expression of the W441C mutation at levels comparable to those of the other pathogenic mutations did not produce development defects, despite causing mild mtDNA depletion in the third instar larval stage. Hence, this line allowed us to study the effects of mutant *d-*mtDNA helicase in adult *Drosophila*. First, we analyzed its effects on lifespan, where we observed a slight decrease in longevity in the W441C flies as compared to those overexpressing wild type *d-*mtDNA helicase (Figure 5A). Despite observing a decrease in mtDNA in the larval stage, mtDNA depletion was not observed in 5, 25 or 50 day-old adults expressing the W441C mutation, arguing that normal mtDNA levels are restored in somatic cells. In contrast, we found a slight increase in the level of mtDNA throughout adult life in the wild type overexpressing line (Figure 5B), an observation consistent with the results reported upon overexpression or Twinkle in mice [11], reinforcing the idea that the mtDNA helicase is limiting for mtDNA replication in animals.

### d-mtDNA Helicase Mutations Decrease Cell Proliferation and Increase Apoptosis in vivo

**Figure 6:**
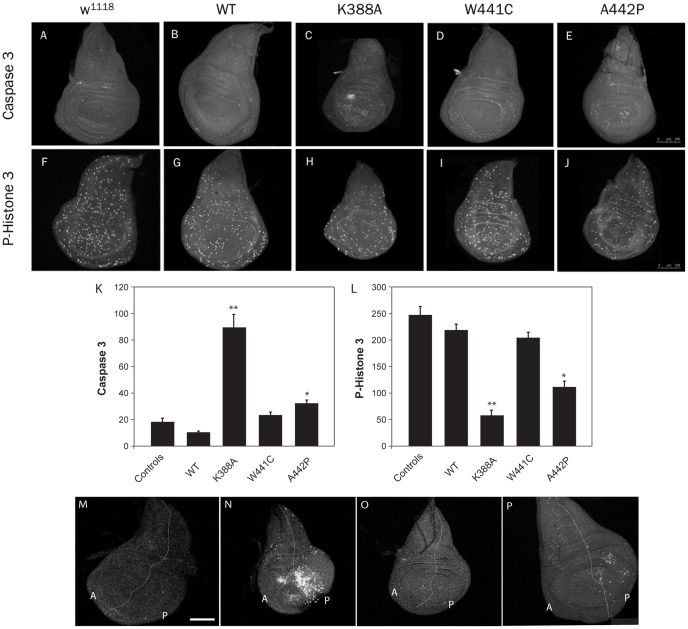
Apoptosis and proliferation levels in wing imaginal discs from third instar larvae. (A–J) Overexpression of the different versions of *d-*mtDNA helicase with the daughterless-Gal4 driver in wing imaginal discs. (A–E) Monoclonal anti-caspase 3 antibody was used for immunocytochemistry to assess apoptotic levels in lines expressing *d-*mtDNA helicase. (F–J) Monoclonal anti-phosphohistone 3 antibody was used for immunocytochemistry to evaluate cell proliferation levels in lines expressing *d-*mtDNA helicase. (K–L) Quantification of apoptosis and cell proliferation levels using a ubiquitous and constitutive driver (*da-GAL4*). Data represent the mean ± s.d., *P≤0.05, **P≤0.005 as compared with controls. (M–P) Overexpression of *d-*mtDNA helicase with the *engrailed* (*en-GAL4*) driver (posterior compartment, P) probed by staining with anti-caspase 3 antibody in third instar larvae’s wing imaginal discs. Overexpression of the K388A mutant shows an increase in apoptosis clusters when compared with the anterior, the control compartment. (M) *w;en-GAL4;UAS helicase^WT^;* (N) *w;en-GAL4;UAS helicase^K388A^;* (O) *w;en-GAL4;UAS helicase^W441C^;* (P) *w;en-GAL4;UAS helicase^A442P^.*

Various mutations in the *d-mtDNA helicase* gene cause a significant depletion of mtDNA. We have shown previously that impairment of mtDNA replication and/or maintenance in *Drosophila* causes mtDNA depletion and an increase in apoptosis [23], [28]. To address this in our *d-mtDNA helicase* mutant lines, we analysed the levels of apoptosis and cell proliferation in the wing imaginal discs of third instar larvae, in lines overexpressing the variants of *d-mtDNA helicase* under the control of different GAL4-drivers. We used anti-caspase3 immunocytochemistry to highlight cells undergoing apoptosis, and anti-phosphohistone antibody to label proliferating cells. Compared with controls, wing imaginal discs from the K338A and A442P mutant lines showed an increase in apoptosis and a decrease in cell proliferation (Figure 6A–L). In contrast, the phenotypes of the lines overexpressing the wild type version and W441C mutant were similar to that of controls (Figure 6K, L). To corroborate these results, we overexpressed the variants of the *d-*mtDNA helicase in the posterior compartment of wing discs using the driver *en-GAL4* and evaluated apoptosis such that the anterior compartment could be used as an internal control with the same cellular background. As shown in Figure 6, we observed an increase in apoptotic clusters in the posterior compartment (Figure 6N,P) as compared to the anterior compartment, suggesting that the elevated levels of apoptosis are due specifically to the documented mitochondrial dysfunction, arguing that mitochondria are key players in this pathway.

## Discussion

A previous study of *d-*mtDNA helicase knockdown in *Drosophila* Schneider cells using RNAi [24] showed cellular phenotypes of slow growth, reduced viability and a 5-fold reduction in mtDNA copy number. Whereas overexpression of the wild type helicase modestly increased mtDNA copy number, overexpression of an active site mutant K338A resulted in a dose-dependent mtDNA depletion, more severe than that observed upon *d-*mtDNA helicase knockdown. Furthermore, whereas overexpression of the A442P mutant significantly reduced mtDNA copy number, overexpression of the W441C mutant did not.

To extend these findings and in consideration of the need to develop tractable animal models to study mitochondrial diseases, we employed the UAS-GAL4 system to examine the effect of the K338A, W441C, and A442P mutations in *d-mtDNA helicase*, and have established a *Drosophila* model of nucleo-mitochondrial communication defect, in a system in which the physiological conditions of the whole organism can be evaluated to understand pathophysiological disorders. We observed a severe decrease in mtDNA copy number and mtDNA-encoded transcripts in the K388A and A442P mutant lines, which caused lethality at the third larval instar and pupal stages, respectively. However, the overexpression of the mutant W441C caused only slight mtDNA depletion without apparent developmental effects.

Interestingly, unlike the findings reported for adPEO patients [29], and in line with the observations in human cells [13], in the *Drosophila* system, mtDNA deletions were not found either in S2 cell lines or in animals. A likely explanation might be that the generation of deleted molecules in *Drosophila* cells is slower than in mammals. Another explication for the findings observed in this and previous studies [13] could be that replication stalling caused by overexpression of mutant versions of Twinkle might induce an increased rate of mtDNA turnover, via mitophagy [12], [30]. The molecular mechanism by which a dominant mutation in mtDNA helicase generates multiple mtDNA deletions is unknown [31], and the accumulation of deleted mtDNA molecules varies widely in different postmitotic tissues in adPEO patients [29], [32]. Similarly, in a transgenic mouse model expressing a pathogenic adPEO duplication in the *Twinkle/C10orf2* gene, mtDNA deletions were detected only after 18 months of life [12] suggesting that mtDNA defects may be a direct consequence of age-associated deterioration of the repair mechanisms (discussed in [13]). Despite of this discrepancy, and in contrast to that observed in human patients, the absence of mtDNA deletions is a common observation in other genetics defects affecting the nDNA-mtDNA intergenomic communication [33], [34], [35].

In adPEO patients, many of the mutations in the *Twinkle* gene map within a small region, which corresponds to the linker region of bacteriophage T7 gp4. The linker region separates the primase and helicase domains and is important for hexamer formation [36]. We found that the A442P mutant displays a dominant negative effect like the helicase active site mutant K338A. Lys^338^ is analogous to Lys^318^ in T7 gp4, and is not essential for T7 gp4 hexamer formation [37], [38]. Thus, the mutant K338A likely forms hexamers with the same efficiency as the wild type polypeptides. However, in the T7 gp4 enzyme, analogous residue A475 in the helicase domain is in close contact with analogous residues R374 and F370 in the linker region, suggesting that it may be required to form stable multimers. The finding that the A475P adPEO variant fractionates mainly as a monomer in gel filtration supports this interpretation for the human enzyme [39]. Likewise, the loss of helicase activity due to hexamer instability could thus explain the phenotype observed in this work in the overexpression of the A442P mutation in flies; whereas hexameric proteins were detected in mitochondrial extracts from Schneider cells, the conformation and/or stability of the oligomers may be compromised.

We found that the overexpression of the wild type form of *d-*mtDNA helicase slightly increases mtDNA copy number in adults, as it does both in Schneider cells and in mice [11], [24], indicating that *d-*mtDNA helicase is modestly limiting for mtDNA replication under physiological conditions. Overexpression of the W441C mutant reduces the mtDNA content in the larval stages, but is recovered in adult cells. This suggests that mtDNA depletion occurs during embryonic and/or larval development but that replacement of mtDNA in postmitotic cells is relatively slow and may not be affected significantly by the W441C mutation, at least during the relatively short life of *Drosophila.* Interestingly, the life span of these animals was slightly reduced as compared to animals overexpressing the wild type form. In the T7 gp4 enzyme, the amino acid orthologous to W441C in flies is not predicted to interact directly with the linker region and in theory, this mutant is able to form hexamers. However, *in vitro* helicase activity of the equivalent W474C mutant in human cells was reduced by 70%, leading to a decrease in mtDNA levels *in vivo* [6], [13], [39]. 2D-PAGE analysis showed that overexpression of this mutant produces an altered pattern of replication intermediates, suggestive of a specific effect in initiation. Furthermore, nucleoid morphology was perturbed in the W474C expressing cells, suggesting a mtDNA segregation defect [13].

The most obvious phenotype in the *Drosophila* lines overexpressing the K388A and A442P mutations is premature death in the larval and pupal stages, respectively, with a decrease in mtDNA copy number as compared with control lines. We explored the effect on mitochondrial function of this mtDNA depletion, and found reduced activity of complex IV, indicating an impairment of OXPHOS function. Increased levels of ROS have been documented to be a direct consequence of OXPHOS impairment [40]. Nonetheless, we found no evidence of an increase of ROS or of oxidative damage in any of the *Drosophila* lines overexpressing the *d-*mtDNA helicase mutants, consistent with observations in adPEO patients [41]. In fact, there are notable differences in comparing the effects produced by other mitochondrial-associated mutations in *Drosophila*. For instance, overexpression of the catalytic core of pol γ, the product of the *tamas* gene, interferes with mtDNA replication leading to mtDNA depletion, OXPHOS defects and an increase in oxidative stress [28]. It is known that the mitochondrial quality control pathway is regulated strictly by various factors [22], such that defective mitochondria may be removed by mitophagy before the accumulation of ROS occurs, suggesting that different alterations in respiratory chain function may affect ROS production in diverse ways.

A direct consequence of the overexpression of the K388A and A442P helicase mutants and likely due to the mtDNA depletion is an increase in apoptosis and a decrease in cell proliferation. We reported previously that silencing or overexpression of genes involved in replication and expression of mtDNA in *Drosophila* increases significantly the number of apoptotic cells [23], [42]. It has also been shown that the mitochondria play a central role in the apoptosis pathway in mammals [17]. Even though nearly all of the proteins involved in cell death pathways are conserved throughout metazoan evolution, the importance of mitochondria in the *Drosophila* apoptotic pathway is both a controversial and an active field of research [18], [19], [20]. *Drosophila* lacking one of the *d-cyt-c* genes was reported to exhibit severely delayed apoptosis in the developing retina [43], and mutations in *d-cyt-c* were found to have reduced caspase activation during spermatid individualization [44]. These and other findings have led to the hypothesis that alterations in mitochondrial morphology might lead to formation of a cytochrome *c*-dependent apoptosome at the mitochondrial surface as part of a feed*-*forward signal in caspase activation [44], [45]. Although the mechanism remains unclear, the results obtained here and in our previous studies [23], [42] allow us to conclude that mitochondrial dysfunction associated with defects in replication and expression of mtDNA induces apoptosis, arguing that an intrinsic mitochondrially-mediated apoptotic pathway is active in *Drosophila*, and offering the possibility to explore its role in pathophysiological processes.

## Materials and Methods

### Fly Stocks and Culture

Flies were raised on standard yeast-glucose- agar medium at 25°C and 65% relative humidity in 12 h light/dark cycles, unless otherwise indicated. To determine the chromosomal location of the transgenes and for the manipulation of transgenic lines, the stock *w*; *CyO*/*If*;*TM6B*/*MKRS* was used. Other GAL4 stock lines used were: *w;;da-GAL4, w;ey-GAL4* and *w;en-GAL4-UAS GFP*, obtained from the Bloomington *Drosophila* Stock Center at Indiana University. For more details of *Drosophila* lines, see Information S1.

### Generation of Transgenic UAS-d-mtDNA Helicase Lines

The constructs used to overexpress the different versions of the *d-mtDNA helicase* gene comprised cDNA fragments from *d-mtDNA helicase* cloned into the pUAST *Drosophila* transformation vector. The *d-mtDNA helicase* insert was obtained as previously described [24]. Recombinant pUAST*- d-mtDNA helicase* constructs were transformed in DH5a competent bacterial cells (Stratagene). Transgenic lines were generated by microinjection of the pUAST- *d-mtDNA helicase* plasmids in *y* ^1^ *w* ^1118^ embryos following standard procedures [24]. Ten independent transgenic lines for each construct were obtained; all of them overexpressed *d-mtDNA helicase*. Each of them carried a single independent insertion in chromosome II or III, which was brought to homozygosis. For more information see Information S1.

### UAS-GAL4 Experiments

*d-mtDNA helicase* fragments were overexpressed using the UAS-GAL4 system described by Brand and Perrimon [23]. The daughterless-GAL4 driver (*da-GAL4*) yields a high and ubiquitous transgenic expression throughout development and was used to induce the overexpression of *d-mtDNA helicase*. The homozygous *da-GAL4* driver was crossed to homozygous UAS lines and the progeny were subjected to study. The same procedure was followed with the *w; en-GAL4* (posterior compartment) driver. Crosses were set up in standard food vials and environmental conditions, and the flies were passed every three days. Additional details on *Drosophila* lines are presented in the Information S1 tables.

### Viability and Longevity Curves of D. Melanogaster

For viability curves, 100 embryos from lines overexpressing the various mutations of *d*-*mtDNA helicase* were collected. Embryos were incubated at 25°C with wet yeast, and the number of animals reaching the developmental stages of third instar larvae, pupae and adults were scored. For longevity curves, 200 adult males were collected from the same lines and placed in a vial containing fresh culture medium. Flies were transferred to fresh medium three times a week, and dead flies were counted every day and a Kaplan-Meier survival curve was performed.

### Real-time RT-PCR

Total RNA was extracted using TRIzol reagent (Invitrogen), and 1 µg was converted into cDNA using the QIAGEN quantitect reverse transcription kit following the manufacturer’s instructions. Genomic DNA was isolated from larvae by standard methods. Real-time PCR was performed using the *7900 HT Fast Real-Time PCR System* (Applied Biosystems), and PCR products were quantified fluorometrically using the *taqman probe* (Applied Biosystems); *d*-*mtDNA helicase*: Dm01809992_g1; *d*-COX I (Fw): TGACTACATCCTCCTGCTCTTTCTT, *d*-COX I (rev): AGCGGATAAAGGTGGATAAACAGTT, FAM probe: TTCCAGCTCCATTTTC; *d*-ND5 (Fw): AGGATTTTTAAAATTAGCTATATTTCATTTATTAACTCATGCTTT, *d*-ND5 (Rev): ACCCCCCTATTAAACGAATATCTTGAGA, FAM probe: CCCAGCACATATAAAC. At least three independent amplifications were performed in triplicates for each transcript, and mean values were normalized to the mean value of the reference rRNA 18S: (Fw): AGCCTGCGGCTTAATTTGACT, (rev): CTTACACACTTATGTTCGACCTGGT and FAM probe: AACACGGGAAAACTT. The PCR amplification program was 95°C for 15 s, 60°C for 1 min, 40 cycles with an initial step of 2 min 50°C and 95°C for 10 min. For mtDNA/nuclear-DNA relative quantification genomic DNA from *Drosophila* third instar larvae was purified and quantified by standard methods. 10 ng of DNA were added to Master Mix-PCR Applied Biosystem. The PCR amplification program was 95°C for 15 s, 60°C for 1 min, 40 cycles with an initial step of 2 min 50°C and 95°C for 10 min. Software used was SDS 2.3 (Applied Biosystems). Assays were performed with two different *taqman* probes: ND5 and 18S.

### Immunocytochemistry in Imaginal Wing Discs

Imaginal discs from third instar larvae were dissected in PBS and fixed with 4% paraformaldehyde in PBS for 20 min at room temperature. They were blocked in PBS containing 1% bovine serum albumin and 0.3% Triton X-100 for 1 h, incubated with the primary antibody overnight at 4°C (1∶50 dilution), washed four times in blocking buffer, and then incubated with the appropriate secondary antibody (1∶200 dilution). for 2 h at room temperature in the dark. Finally, they were washed and mounted in Vectashield (Vector Laboratories). Primary antibodies used were: rabbit anti-phosphohistone 3 (Sigma-Aldrich) and rabbit anti-caspase 3 (Cell Signaling Technology). Secondary antibodies were coupled to the fluorochromes Alexa Fluor 647 (Invitrogen) or Alexa 555 (Invitrogen). In addition, the nucleus was stained with TO-PRO 3 iodide (Invitrogen) and actin with Phalloidin-TRITC (Sigma-Aldrich). Preparations were imaged with a Leica TCS SP2 laser-scanning microscope (Heidelberg, Germany). The number of apoptotic cells was measured using analySIS FIVE software (Olympus Digital Imaging Solutions).

### Oligonucleotides

All oligonucleotides were purchased from Isogen (the Netherlands) and Sigma-Aldrich. All sequences are given in the 5’ to 3’ direction. Probes used for Southern and Northern hybridization were PCR products obtained from the amplification of *Drosophila* nuclear and mitochondrial DNAs using the following primers: *d-mtDNA helicase* (dir): ATGAGACGCGCCGGTTTAA; *d-mtDNA helicase* (rev): TCAGTTCTCGGATGGCGTCT; *d*-histone (dir): ACACACGGAACACGAATGCTCG; *d*-histone (rev): AGCGAAGCCAAAGCCTGTAGTAGC. Oligonucleotides used for quantitative PCR (qPCR) are as follows: *d-mtDNA helicase*: TaqMan Gene Expression Assays Dm01809992_g1; *d*-COX I (dir): TGACTACATCCTCCTGCTCTTTCTT, *d*-COX I (rev): AGCGGATAAAGGTGGATAAACAGTT, FAM probe: TTCCAGCTCCATTTTC; *d*-ND5 (dir): AGGATTTTTAAAATTAGCTATATTTCATTTATTAACTCATGCTTT, *d*-ND5 (rev): ACCCCCCTATTAAACGAATATCTTGAGA, FAM probe: CCCAGCACATATAAAC. For more details see Information S1.

### Quantitative RT-PCR Assays

∼10 frozen larvae from each genotype were used for RNA extraction with TRIzol reagent (Invitrogen). Total RNA from each sample (2 µg) was reverse transcribed into cDNA using oligo(dT) as a primer and the SuperScript III First-strand Synthesis System for RT-PCR (Invitrogen), according to the manufacturer’s instructions. The gene encoding the 18S rRNA was used as a normalizing internal control [25]. Exon-specific oligonucleotide primers for the *d-mtDNA helicase* were designed with Primer3 software. Real time PCRs were performed in a Rotor Gene thermocycler (Corbett Research) using Taqman probes (Applied Biosystems) as double-stranded DNA binding dye and under the following conditions: 50°C for 2 min, 95°C for 10 min; 40 cycles (95°C for 15 sec and 60°C for 1 min annealing/extension. Each sample contained 1X Taqman probe, 0.2 mM dNTPs, 1X enzyme buffer, 4 mM MgCl_2_, 0.5 units of AmpliTaq Gold DNA polymerase UP, 0.3 µM of each oligonucleotide, and 4 µl of a 1/20 dilution of the reverse transcribed products in a final volume of 20 µl. Three separate samples were collected from each genotype and triplicate measurements were performed.

### Immunoblotting

Protein extracts (30 µg) obtained from mitochondria purified by differential centrifugation were fractionated by 10.5% SDSPAGE and transferred to nitrocellulose filters. Filters were pre-incubated for 1 h with 5% skim milk in TBS, followed by incubation for 1 h with the corresponding primary antibody. Polyclonal antibody against *d-mtDNA helicase* was used as previously described [24]. As a loading control, an antibody against *Drosophila* VDAC-porin was used at a 1∶2000 dilution.

### DNA Extraction and Southern Blotting

Aliquots of 20 frozen third instar larvae were ground in 800 µl of homogenization solution that contained 10 mM Tris-HCl, pH 7.5, 10 mM EDTA, 60 mM NaCl, 0.5% SDS. Precipitated material was removed by centrifugation and the supernatant fractions were extracted once with phenol/ chloroform (1∶1), treated with 10 µg/ml RNase A at 37°C for 15 min, re-extracted with phenol/ chloroform (1∶1), and precipitated with 2 volumes of ethanol. DNA precipitates were washed with 80% ethanol and resuspended in 50 µl of H_2_O. Southern analysis was performed as described previously [25].

### Mitochondrial Preparations

For enzymatic measurements, aliquots of fresh third instar larvae were ground in buffer containing 250 mM sucrose, 2 mM EDTA, 100 IU/liter heparin, 10 mM Tris-HCl, pH 7.4 and mitochondria were isolated by differential centrifugation; purified mitochondria were sonicated for 6 s at 4°C, frozen and thawed. Protein determination was performed using the DC Protein Assay kit (Bio-Rad).

### OXPHOS Activities

Mitochondrial OXPHOS complex and citrate synthase activities were measured as described previously [23]. Incubation was at 30°C except for complex IV, which was measured at 38°C. Enzyme activities are expressed in nanomoles of substrate catalyzed per minute and per citrate synthase activity.

### Reactive Oxygen Species (ROS) Production

Mitochondrial superoxide was assessed using the fluorogenic dye MitoSOX (Molecular Probes) and dihydroethidium (Invitrogen). 20 wing imaginal discs from third instar larvae were dissected in 1X PBS. Cell dissociation of wing discs was performed as described previously [32] and incubated with 5 mM MitoSox in this medium for 10 min at 37°C in the dark. The cell suspension was analyzed by flow cytometry (BD Biosciences FACS Vantage SE) and data were processed with the BD FACS Diva and Cell Quest Pro software (Becton Dickinson). Alternatively, 20 wing imaginal discs from third instar larvae were dissected and incubated *in vivo* with the dye as previously described [26]. Preparations were imaged with a Leica TCS SP2 laser-scanning microscope (Heidelberg, Germany).

### Statistical Analysis

Statistical analysis was performed with the Prism 4.03 software (GraphPad Software). The one-way ANOVA test and the Bonferroni post-test were used to determine the statistical significance of the results.

## Supporting Information

Information S1 **Supporting tables.**
(DOCX)
