# Supplementary material for: Modeling Pathogenic Mutations of Human Twinkle in Drosophila Suggests an Apoptosis Role in Response to Mitochondrial Defects
Source: PLoS One. 2012 Aug 28;7(8):e43954. doi: 10.1371/journal.pone.0043954 (PMC3429445; doi:10.1371/journal.pone.0043954)
Supplement: Information S1 — Supporting tables. (DOCX) [file pone.0043954.s001.docx]

**Supplemental information**

**Supplemental tables**

**Table S1.** Control lines and balancer stock.

| **Name used in main text** | **Official or proposed symbol/genotype in Flybase** | **Original reference and source** | **Reference** | **Type of resource** |
| --- | --- | --- | --- | --- |
| yellow white | *y^1^,w^1118^;;* | R. Garesse | - | Control line |
| w^1118^ | *w^1118^;;* | R. Garesse | - | Control line |
| Marked/Balanced stock | *w; Cyo/If; TM6,Tb/MKRS* | R. Garesse | - | Marked/Balanced stock |

**Table S2.** Gal4 Drivers.

| **Name used in main text** | **Official or proposed symbol/genotype in Flybase** | **Original reference and source** | **Reference** | **Type of resource** |
| --- | --- | --- | --- | --- |
| *daugtherless*-GAL4 | *w^1118^;; P{da-GAL4.w-}3* | Bloomington Stock Centre nº 8641 | FlyBase, (1992-) | Gal4 driver line |
| *engrailed*-GAL4 | P{en2.4-GAL4}e16E | Bloomington Stock Centre nº 25752 | - | Gal4 driver line |

**Table S3.** UAS transgenic lines.

| **Name used in main text** | **Official or proposed symbol/genotype in Flybase** | **Original reference and source** | **Reference** | **Type of resource** |
| --- | --- | --- | --- | --- |
| UAS - WT | *w;;UAS-helicase WT/(TM3,Sb)*  *w;UAS-helicase WT/(Cyo)* | This paper | This paper | Transgenic construct |
| UAS - 388 | *w;;UAS-helicase 388/(TM3,Sb)*  *w;UAS-helicase 388/(Cyo)* | This paper | This paper | Transgenic construct |
| UAS - 441 | *w;;UAS-helicase 441/(TM3,Sb)*  *w;UAS-helicase 441/(Cyo)* | This paper | This paper | Transgenic construct |
| UAS - 442 | *w;;UAS-helicase 442/(TM3,Sb)*  *w;UAS-helicase 442/(Cyo);* | This paper | This paper | Transgenic construct |

**Table S4**. Constructs.

| **Name used in main text** | **Official or proposed symbol** | **Original reference and source** | **Type of resource** |
| --- | --- | --- | --- |
| pGEMT-Easy | pGEMT-Easy | Promega | Cloning of PCR products |
| pMT/Hy | pMK33/pMtHy | Koelle, M. (1989). | Expression vector |
| pUAST | pUAST | Brand, A.H., Perrimon, N. (1993). | Transgenic vector |
| pUAST-*dTwinkle* WT | pUAST-*dTwinkle* WT | This paper | Transgenic construct with the *d-mthelicase* wild type |
| pUAST-*dTwinkle* 388 | pUAST-*dTwinkle* K388A | This paper | Transgenic construct with the *d-mthelicase* K388A mutation |
| pUAST- *dTwinkle* 441 | pUAST-*dTwinkle* W441C | This paper | Transgenic construct with the *d-mthelicase* W441C mutation |
| pUAST-*dTwinkle* 442 | pUAST-*dTwinkle* A442P | This paper | Transgenic construct with the *d-mthelicase* A442P mutation |

**Table S5.** Primers.

| **Name used in main text** | **Sequence** | **Type of resource** | **mT** | **Reference** |
| --- | --- | --- | --- | --- |
| Dm mt genome A Dir | GCTGGAATTGCTCATGGTGGA | Long PCR mtDNA Dir | 64ºC | (Yui et al. 2003) |
| Dm mt genome A Rev | AGGGTGATTTGAGTGTGTAGAC | Long PCR mtDNA Rev | 64ºC | (Yui et al. 2003) |
| Dm mt genome B Dir | GTCTACACACTCAAATCACCCT | Long PCR mtDNA Dir | 64ºC | (Yui et al. 2003) |
| Dm mt genome B Rev | TAGGGTGAGATGGTTTAGGACT | Long PCR mtDNA Rev | 64ºC | (Yui et al. 2003) |
| Dm mt genome C Dir | TGTGAATAATAGCCCCAGCACA | Long PCR mtDNA Dir | 64ºC | (Yui et al. 2003) |
| Dm mt genome C Rev | GTTGAATATGGGCAGGTGTTAC | Long PCR mtDNA Rev | 64ºC | (Yui et al. 2003) |
| Dm mt genome D Dir | GTAACACCTGCCCATATTCAAC | Long PCR mtDNA Dir | 64ºC | (Yui et al. 2003) |
| Dm mt genome D Rev | GCCAGCAGTCGCGGTTATAC | Long PCR mtDNA Rev | 64ºC | (Yui et al. 2003) |
| Helicase Dir | ATGAGACGCGCCGGTTTAAT | *d*-mtHelicase Dir | 60ºC | This paper |
| Helicase Rev | TCAGTTCTCGGATGGCGTCT | *d*-mtHelicase Rev | 62ºC | This paper |
| pUAST Dir | ccaattatgtcacaccacaga | Sequencing mthelicase clones | 58ºC | This paper |
| pUAST Rev | ctgcaactactgaaatctgcc | Sequencing mthelicase clones | 62ºC | This paper |
| 1659 | ACACACGGAACACGAATGCTCG | Histone cluster probe Dir | 68ºC | This paper |
| 1660 | AGCGAAGCCAAAGCCTGTAGTAGC | Histone cluster probe Rev | 70ºC | This paper |
